# Perforated Subhepatic Appendicitis Mimicking Hepatobiliary Pathology: A Case Report and Narrative Literature Review

**DOI:** 10.7759/cureus.111437

**Published:** 2026-06-24

**Authors:** Christina Van Hemmen Kon, Jenna Phillips, Niamh Varrallo, Arravintha Gobinathan, David Marshall, Jeffrey P Snow, Jordan J Ditchek, Gary Schwartz

**Affiliations:** 1 Allopathic Medicine, Nova Southeastern University Dr. Kiran C. Patel College of Allopathic Medicine, Fort Lauderdale, USA; 2 Pathology, Memorial Healthcare System, Hollywood, USA; 3 General Surgery, Memorial Healthcare System, Hollywood, USA; 4 Medical Education, Nova Southeastern University Dr. Kiran C. Patel College of Allopathic Medicine, Fort Lauderdale, USA; 5 Orthopedic Surgery, Nova Southeastern University Dr. Kiran C. Patel College of Allopathic Medicine, Fort Lauderdale, USA

**Keywords:** atypical appendicitis, cecal maldescent, congenital gut anomaly, diagnostic delay, intestinal malrotation, mobile cecum, perforated appendicitis, right upper quadrant pain, subhepatic appendicitis, subhepatic cecum

## Abstract

Subhepatic appendicitis is a particularly rare anatomical variant presenting with atypical right upper quadrant (RUQ) pain mimicking hepatobiliary pathology. This mimicry frequently leads to diagnostic delay and subsequent increased risk of complications.

A 41-year-old man presented to the Emergency Department with acute RUQ pain, radiating to the back and shoulder, accompanied by constitutional and gastrointestinal symptoms. RUQ ultrasound visualized no gallbladder abnormalities; subsequent CT scan revealed a dilated appendix with a rounded tip and possible presence of an abscess. Initial surgical intervention was attempted laparoscopically; however, conversion to an open appendectomy was required due to the complex anatomy, including a subhepatic cecum adhered to the retroperitoneum, and intraoperative bleeding. The cecal caput was divided to preserve the ileocecal valve. Pathological assessment was consistent with appendicitis with rupture due to postinflammatory changes. The postoperative course was largely uncomplicated with transient ileus, and the patient was discharged on postoperative day 3.

Although subhepatic appendicitis is rare, it should be considered when patients present with RUQ pain and hepatobiliary assessment proves negative. In case of a nondiagnostic ultrasound, CT imaging should be employed early in the diagnostic exploration. As conversion from a laparoscopic to an open approach may be warranted in these cases, the surgical team should be vigilant when encountering such a patient.

## Introduction

Acute appendicitis is the most common abdominal surgical emergency in the world, with an annual incidence of approximately 100 cases per 100,000 adults [1]. The classic symptoms associated with appendicitis include fever, leukocytosis, anorexia, nausea and vomiting, and abdominal pain. The pain classically migrates from a diffuse periumbilical location to the right lower quadrant (RLQ), as the inflammation expands to the surrounding parietal peritoneum in the ileocecal region. Although over 50% of patients present with this classic pattern of migrating pain, there are some instances where patients indicate pain in differing locations [1].

During the first trimester of uncomplicated fetal development, the cecum is located in a subhepatic position [2]. With normal intestinal rotation, the cecum migrates from a subhepatic position in the first trimester to the right lumbar region in the second and third trimesters, before ultimately descending into the right iliac fossa [2]. Abnormalities during this rotation or failure of the subsequent descent can result in the persistence of a subhepatic appendix.

In such cases, patients often present with right upper quadrant (RUQ) pain rather than the more classic RLQ pain, as the appendix is located below the liver in a superior cecal position, mimicking hepatobiliary pathology such as cholecystitis. A subhepatic appendicitis is rare, with a subhepatic cecum occurring at rates of 0.3%-0.5% of live births [3]. The appendix is most commonly located in the retrocecal position (32.1% of the population), followed by pelvic (28.5%) and ileal (14.5%) positions, underscoring the relative rarity of a subhepatic position [4]. The incidence of subhepatic appendicitis was approximately 0.08% based on a single-center retrospective case series of over 7,200 appendicitis cases [5].

The rarity of this anatomic variant, combined with the subsequent atypical presentation of appendicitis, can complicate timely diagnosis. Symptoms in these cases can closely mimic hepatobiliary conditions, which typically present with fever, nausea, RUQ pain, and a positive Murphy's sign, increasing diagnostic complexity [6]. Additionally, standard scoring systems to aid decision-making, such as the Alvarado score, and initial ultrasound imaging may be less effective.

Increased complexity can result in diagnostic delay, thereby increasing the risk of complications, as the persistence of symptoms for more than 24 hours is a known risk factor for perforation [1]. Perforated appendicitis is associated with increased risk of abscess formation, hospital readmission, and is associated with a sextupled mortality rate compared to nonperforated cases [7,8]. This case report discusses an uncommon perforated subhepatic appendicitis that initially mimicked hepatobiliary pathology, highlighting the diagnostic challenges, management, and implications of this variant.

## Case presentation

A 41-year-old man presented to the Emergency Department (ED) with RUQ abdominal pain, diarrhea, nausea, and vomiting that had begun two days before presentation. The pain was described as starting in the RUQ and radiating to both the back and shoulder without radiation to the RLQ. Additionally, he experienced a self-reported low-grade fever and chills. His medical history was unremarkable. His vital signs were unremarkable with a temperature of 98.2°F, heart rate of 112 bpm, blood pressure of 112/72 mm Hg, respiratory rate of 14 breaths/minute, and oxygen saturation of 100% on room air. Upon physical examination, the patient was alert and oriented, and did not appear in acute distress. His head and eyes were atraumatic and normocephalic with clear corneas. No abnormalities in the ears were noted, and a full range of motion of the neck was noted. The lungs were clear to auscultation, and examination of the heart and pulses showed regular rate and rhythm. The abdomen was soft, but RUQ abdominal tenderness with localized guarding was noted. No edema or restriction of movement was found in the extremities, and there were no motor or sensory deficits. Laboratory evaluation was performed, and results are shown in Table 1.

**Table 1:** Laboratory values emergency department (CBC, CMP, coagulation panel, and urinalysis) ESR and CRP were not ordered BUN: blood urea nitrogen; GFR: glomerular filtration rate; AST: aspartate aminotransferase; ALT: alanine aminotransferase; PT: prothrombin time; INR: international normalized ratio; WBC: white blood cell; RBC: red blood cell; Hgb: hemoglobin; Hct: hematocrit; MCV: mean corpuscular volume; MCH: mean corpuscular hemoglobin; MCHC: mean corpuscular hemoglobin concentration; RDW: red cell distribution width; MPV: mean platelet volume; CBC: complete blood count; CMP: complete metabolic panel; ESR: erythrocyte sedimentation rate; CRP: C-reactive protein; H: high; L: low

Laboratory	Result	Reference range	Units
Chemistry			
Sodium	140	135-145	mEq/L
Potassium	4.3	3.5-5.2	mEq/L
Chloride	101	95-110	mEq/L
Carbon dioxide	27	19-34	mEq/L
BUN	12	6-22	mg/dL
Creatinine	0.9	0.43-1.13	mg/dL
Estimated GFR	>90	≥90	mL/min/1.73 m²
Glucose	96	70-110	mg/dL (fasting)
Calcium	9	8.4-10.2	mg/dL
Corrected calcium	8.3 (L)	8.4-10.2	mg/dL
Total bilirubin	0.8	0.1-1.2	mg/dL
AST	19	10-40	U/L
ALT	17	10-60	U/L
Alkaline phosphatase	40	20-130	U/L
Total protein	8	5.5-8.7	g/dL
Albumin	4.3	3.2-5.0	g/dL
Coagulation			
PT	13.4 (H)	10-12.8	seconds
INR	1.2 (H)	0.8-1.1	ratio
Hematology			
WBC	9.1	4.0-10.5	×10³/µL
RBC	4.17 (L)	4.63-6.08	×10⁶/µL
Hgb	12.4 (L)	13.5-17.5	g/dL
Hct	37.4 (L)	40.1-51.0	%
MCV	89	79.0-92.2	fL
MCH	29.7	25.7-32.2	pg
MCHC	33.4	32-36	g/dL
RDW	12.1	11.5-14.5	%
Platelet count	288	150-400	×10³/µL
MPV	9.4	9.4-12.4	fL
Nucleated RBC %	0	0.0-0.2	%
Immature granulocyte %	0.1	0.0-0.4	%
Basophils %	0.2	0.2-1.2	%
Immature granulocyte (count)	0.01	0.0-0.3	×10³/µL
Absolute neutrophils	6.14 (H)	1.78-5.38	×10³/µL
Segmented neutrophils	67.9	34.0-67.9	%
Lymphocytes	19.1 (L)	21.8-53.1	%
Lymphocytes (count)	1.73	1.32-3.57	×10³/µL
Monocytes	11.4	5.2-12.2	%
Monocyte (count)	1.03 (H)	0.3-0.82	×10³/µL
Eosinophils	1.3	0.8-7.0	%
Eosinophils (count)	0.12	0.04-0.54	×10³/µL
Basophils (count)	0.02	0.01-0.08	×10³/µL
Nucleated RBC (count)	0	0-0.18	×10³/µL
Urine			
Color	Yellow	Yellow (pale-dark)	-
Appearance	Clear	Clear	-
pH	6	5.0-9.0	-
Specific gravity	>1.030 (H)	1.005-1.030	-
Protein	20 (Trace)	Negative - Trace	mg/dL
Ketones	100 (3+) (H)	Negative - Trace	mg/dL
Blood	Negative	Negative - Trace	mg/dL
Nitrite	Negative	Negative	-
Bilirubin	Negative	Negative	mg/dL
Urobilinogen	Normal	Negative	mg/dL
Leukocyte esterase	Negative	Negative - 25	Leu/µL
Glucose	Normal	Normal - Trace	mg/dL

These findings showed a white blood cell count in the normal range (9.1 x 10³/µL) with neutrophilia (absolute neutrophils 6.14 x 10³/µL), lymphopenia (19.1%), and monocytosis (1.03 x 10³/µL), suggesting an underlying inflammatory process. Mild normocytic anemia was seen with a hemoglobin of 12.4 g/dL, a hematocrit of 37.4%, and a mean corpuscular volume (MCV) of 89 fL. The coagulation panel showed a mildly prolonged prothrombin time (PT) at 13.4 seconds, with an international normalized ratio (INR) of 1.2. Corrected calcium was slightly low at 8.3 mg/dL. Urinalysis indicated dehydration and poor oral intake, likely due to the patient's complaints of nausea and vomiting. These lab findings were in the context of normal liver enzymes, renal function, bilirubin, electrolytes, and platelet counts. The patient's Alvarado Score was estimated at 1-2: points for nausea/vomiting and borderline left shift at ~67.5% neutrophils, which did not meet the ≥75% threshold. No points were given for the fever, as the measured temperature did not meet the fever threshold of 99.5°F.

RUQ ultrasound revealed no gallbladder abnormalities and was followed by an abdominal CT scan due to ongoing clinical suspicion. Additionally, a chest X-ray was performed to rule out an acute cardiopulmonary process and was negative. CT revealed no abnormalities in the biliary and hepatic system but identified a dilated appendix in the RUQ space (Figure 1), measuring up to 1.5 cm. Additionally, some retroperitoneal loss of the fat plane was noted in the periappendiceal region, thus raising concerns for a possible appendiceal abscess (Figure 2).

**Figure 1:**
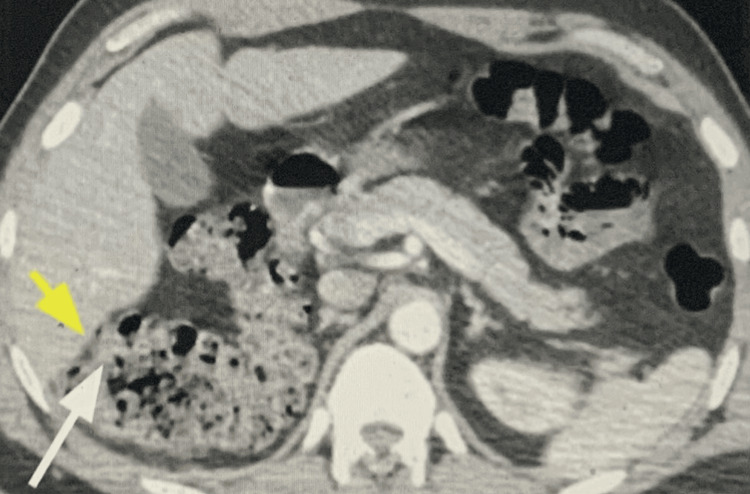
CT image of appendiceal subhepatic location Axial image from a contrast-enhanced CT scan of the abdomen demonstrates the cecum positioned relatively superiorly in the right abdomen as an anatomical variant, with its tip (white arrow) directly laterally less than 1 mm from the undersurface of the right hepatic lobe (yellow arrow)

**Figure 2:**
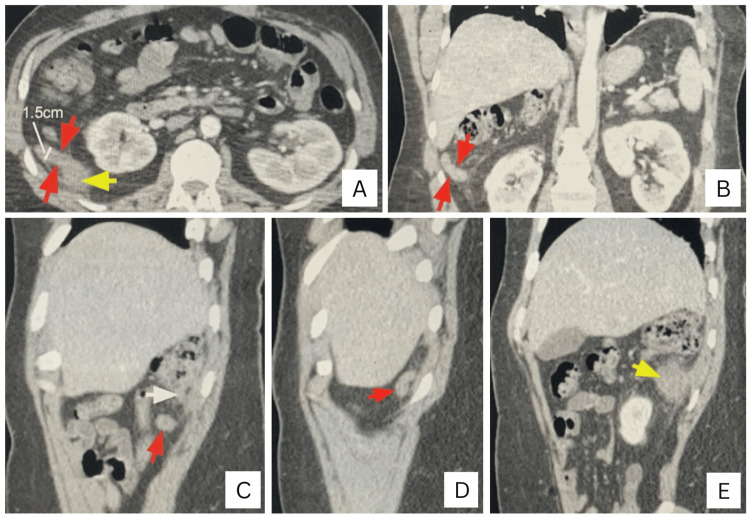
Preoperative CT scan of the abdomen Axial (A), coronal (B), and sagittal (C-E) images from a contrast-enhanced CT scan of the abdomen demonstrate a dilated appendix measuring 1.5 cm in diameter (red arrows), which contains no air and has a thickened, enhancing wall. Note the associated inflammation causing mild stranding in the periappendiceal fat and thickening of adjacent fascial planes. The base of the appendix arises from a superiorly positioned cecum (white arrow in C). An associated phlegmon (yellow arrows in A and E) is positioned in the deep posterolateral aspect of the right abdomen, near the upper pole of the right kidney

General surgery was consulted, and surgical intervention was indicated. A laparoscopic appendectomy was initially attempted; however, the cecum was found in the RUQ with the terminal ileum attached to the right gutter along the white line of Toldt. The atypical anatomy complicated visualization of the appendix and differentiation of the appendiceal tip from the cecum. The surgeon traced the transverse colon to the RUQ to locate the cecum and deduce the location of the appendiceal tip. Upon manipulation of the appendix, it was found adherent to the retroperitoneum, and lifting it caused purulent drainage and bleeding. Therefore, the decision was made to switch to an open laparotomy via a midline incision. This allowed the surgeon to use their fingers to pinch the appendix off the retroperitoneum and bring the cecum and appendix into the surgical field.

Upon visualization, it became clear that the inflammation had spread into the caput of the cecum, with a perforation originating in the appendix extended into the midportion of the cecum. It was determined that the appendix could be resected while preserving the ileocecal valve by dividing the cecal caput. The mesoappendix was divided with an Enseal device (Ethicon, Cincinnati, OH), and the caput was freed, followed by division of the terminal ileal fat pad for mobilization. The caput of the cecum was divided with three firings of a gastrointestinal anastomosis stapler without compromising the terminal ileum. The appendix was then removed, the cecum was repositioned in the anatomic RLQ location, and the bowel was returned to its anatomical position. Estimated blood loss was 50 cc, and a Jackson-Pratt (JP) drain (Cardinal Health, Dublin, OH) was placed due to the presence of an abscess and the initial intraoperative bleeding. Layered closure was achieved with a double-stranded polydioxanone suture, followed by interrupted 0 Vicryl sutures in the subcutaneous fat, interrupted 3-0 Vicryl sutures superficially, and a running 4-0 Monocryl subcuticular closure.

The histopathology findings were consistent with appendiceal rupture and subsequent postinflammatory reaction, confirming the diagnosis of acute appendicitis. The tip of the appendix was adhered to the cecal serosa. Perforation was not grossly identified on the appendiceal wall (Figure 3).

**Figure 3:**
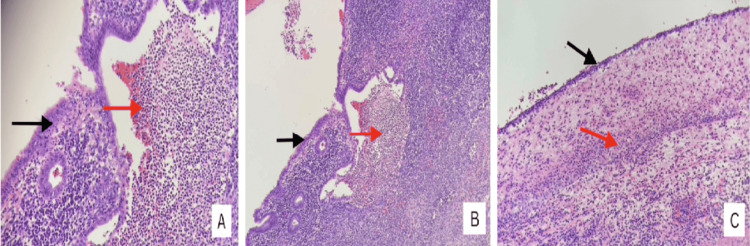
Histopathology of the appendix Histopathology H&E stains (A) ×100 (objective ×10, eyepiece ×10), (B) ×50 (objective ×5, eyepiece ×10), and (C) ×50 (objective ×5, eyepiece ×10) all demonstrate the appendix mucosa with acute inflammation, with predominance of neutrophils (red arrows), consistent with acute appendicitis. The epithelial lining is also demonstrated (black arrows) H&E: hematoxylin and eosin

The postoperative course was notable for ileus on postoperative day (POD) 1 and loose bowel movements on POD 2, both of which resolved before discharge. There were no major complications; the JP drain output was 50 cc, the patient remained afebrile, and the white blood cell (WBC) remained within normal limits at 10.3 x 10³/µL. The patient was discharged on POD 3 with a scheduled telehealth follow-up in three weeks. The timeline of the patient's clinical course from symptom onset through discharge is seen in Figure 4.

**Figure 4:**
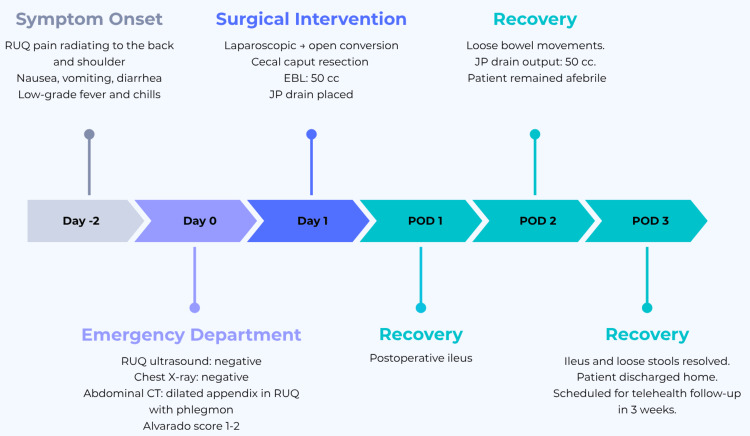
Timeline of the patient’s clinical course from symptom onset through discharge RUQ: right upper quadrant; JP: Jackson-Pratt; EBL: estimated blood loss; POD: postoperative day

## Discussion

Atypical presentations of appendicitis, particularly the subhepatic location, pose significant diagnostic challenges and can frequently be mistaken for alternate pathology. The classical presentation involves periumbilical pain that migrates to the RLQ and is associated with a variety of specific clinical signs and maneuvers, allowing for a generally clear and straightforward diagnosis [1]. McBurney's point tenderness sensitivity varies, as it depends on both the anatomical position of the appendix and the degree of peritoneal involvement [1]. In female patients, delayed diagnosis tends to be more frequent as symptoms can be falsely attributed to gynecologic conditions [9]. When patients present with RUQ pain radiating to the back, alongside fever and gastrointestinal symptoms, it can prove difficult to establish the necessary rapid diagnosis, carrying a higher risk of perforation. Prior reports have demonstrated that the symptoms of subhepatic appendicitis are often initially attributed to hepatobiliary or gastrointestinal disease [6].

Due to the high prevalence of appendicitis, a variety of clinical scoring systems have been outlined to help distinguish it from other causes of abdominal pain [7]. The Alvarado score is one of the most frequently used scoring systems and incorporates considerations regarding a patient's history, laboratory values, and physical exam. The sensitivity of the Alvarado score is generally high, with a recent study reporting sensitivity and specificity of 94.62% and 87.80%, respectively [10]. The scoring ranges from 0 to 10, with lower scores (≤4) suggesting low risk, scores of 5-6 indicating intermediate risk, and scores ≥7 indicating a high probability of appendicitis [7]. Based on these criteria, this patient’s estimated Alvarado score is approximately 1-2. This score is based on the absence of several important criteria, including migration of pain to the RLQ, anorexia, RLQ tenderness, rebound pain, temperature of ≥ 99.5°F, and leukocytosis. The patient did, however, receive points due to their nausea and vomiting, and borderline left shift (67.5% vs. >75% neutrophils). Using this scoring system, the patient would be classified as low risk for appendicitis, and imaging could have been deferred, depending on the clinician's judgment. As such, this case highlights an important limitation of these scoring systems that clinicians should be cognizant of. While these scores are useful and generally accurate, they may not adequately account for atypical presentations. Reliance on the Alvarado score alone could have led to premature discharge and missed diagnosis of perforated appendicitis.

During early fetal development, the midgut typically rotates about 270° counterclockwise around the superior mesenteric artery, followed by descent of the cecum and appendix into the RLQ, resulting in localization of the small and large intestine to their anatomical positions [11]. Anomalies during intestinal rotation, such as malposition of the cecum, can result in atypical clinical presentations of appendicitis, as in this case, which are more difficult to recognize and diagnose [2]. Anatomical variations should be considered in patients presenting with abdominal symptoms to ensure prompt diagnosis, proper imaging interpretation, and operative planning.

Laboratory evaluation in the ED showed a normal WBC count with neutrophilia (absolute neutrophils 6.14 x 10^3^/μL), lymphopenia (19.1%), and nonspecific monocytosis (1.03 x 10^3^/μL). These labs indicate an acute inflammatory process notwithstanding the normal WBC. Inflammation in the context of a normal WBC can be observed in early intra-abdominal inflammatory processes, where the WBC count has not yet risen [12,13]. This highlights the importance of considering an inflammatory process even when the WBC count is not elevated. Additionally, the patient presented with mild normocytic anemia (hemoglobin 12.4 g/dL, hematocrit 37.4%, MCV 89 fL), raising suspicion for an acute inflammatory or infectious process [14]. The PT was somewhat prolonged at 13.4 seconds, with an INR of 1.2 in the context of normal liver enzymes and bilirubin, pointing toward a mild prolongation of the extrinsic coagulation pathway due to an early inflammatory process rather than hepatic origin [15]. Slight hypocalcemia (corrected to 8.3 mg/dL) with an albumin of 4.3 g/dL in the context of normal renal function is likely due to acute inflammation rather than an underlying renal or metabolic origin. Furthermore, combining these findings with normal hepatobiliary labs is feasibly due to an acute inflammatory origin outside the biliary system [16].

Classical presentations of appendicitis are diagnosed using imaging, with ultrasonography commonly utilized as the first-line option, particularly due to its lack of ionizing radiation, accessibility, and relatively high specificity [17]. However, atypical anatomical presentations of appendicitis can prove to be more complicated. This can reduce the sensitivity of ultrasounds due to reduced visualization and possibly cause delays in management [7]. For this patient, considering the initial concern for pathology of hepatobiliary origin, a RUQ ultrasound was performed and did not reveal any hepatobiliary abnormalities. As the imaging was focused on visualizing the hepatobiliary structures, the appendix was not assessed during this initial study. Consequently, the initial suspicion for hepatobiliary pathology, alongside inconclusive imaging, resulted in a diagnostic delay. A CT scan was found to be more effective in aiding the diagnostic process for this case, as it revealed an inflamed appendix in the RUQ. This aligns with recent studies displaying the increased sensitivity of CT imaging compared to ultrasounds, particularly in cases of atypical appendix presentations [18,19]. Confirmation of the presence of a subhepatic appendix occurred intraoperatively. This is commonly done when preoperative imaging is inconclusive, as it requires identification and tracing of the bowel to locate the appendix [7].

Laparoscopic appendectomy is generally the preferred surgical approach for uncomplicated appendectomies due to its association with decreased postoperative pain, shorter length of hospital stay, and faster recovery time compared to an open appendectomy [20]. However, in patients with subhepatic appendicitis, the surgical approach can vary based on the complexity of the particular case. Surgical decision-making takes into account that the visualization of the anatomy can be more challenging, that a higher degree of inflammation and perforation may be present, and that there is an increased risk of complications, and varies on a case-by-case basis [7,17]. In this case, the onset of bleeding resulting in limited visibility guided the decision to convert the surgery to an open laparotomy via a midline incision, demonstrating the need for flexibility in surgical approach.

Both open and laparoscopic appendectomies are widely used and depend on the progression of appendicitis and the ability to visualize structures. Conversion from laparoscopic to open appendectomy occurs in approximately 2%-8% of cases, and the combination of atypical subhepatic positioning, retroperitoneal adherence, and perforation placed this patient at higher risk for conversion [21-23]. Management of retroperitoneal appendiceal perforation brings along additional challenges, such as considerations regarding sepsis prevention and abscess drainage that need to be made [7,8]. Regarding this case, adhesions were present along the right gutter and white line of Toldt, requiring careful dissection of both adhesions and peritoneal attachments. In atypical appendicitis, as in this case, which required extension of the resection into the cecum, preservation of the ileocecal valve is preferred when surgically feasible [24]. Preservation may support normal postoperative bowel function and recovery, and was ensured by dividing and limiting resection to only the inflamed portion of the cecal caput [25]. Surgical drains may be placed in severe cases of perforated appendicitis to allow for evacuation of residual intra-abdominal fluid and reduce the risk of postoperative abscess formation, which has been found to occur in up to 20%-25% of cases [26].

Despite its rarity, the prognosis for subhepatic appendicitis is generally favorable when the diagnosis is made promptly. Complications following appendicitis are relatively uncommon overall, with recent studies reporting postoperative complications in 5.5% of cases; surgical site infections were the most common, accounting for 75% of these complications [27]. However, cases of complicated appendicitis have been associated with significantly higher rates of surgical complications, longer hospital stays, and increased postoperative morbidity [9]. This highlights why early recognition and appropriate surgical intervention are important to prevent complications. This case presentation allows further insight into this rare anatomical variant with clear imaging and operative correlation, potentially aiding diagnosis and treatment of similar cases. This case, as well as the performed literature review, emphasizes the importance of clinical consideration of appendicitis even in more atypical presentations such as RUQ pain.

Literature review

A literature search was conducted using the PubMed database with a timeframe containing articles from 2005 to 2025. The search strategy included combinations of the following keywords: “appendicitis”, “atypical presentation”, “unusual symptoms”, and “diagnostic delay”. The authors collected and reviewed 21 papers related to atypical presentation of appendicitis. Inclusion criteria consisted of peer-reviewed articles, published in English, that described atypical presentations of appendicitis or associated diagnostic challenges. Case reports and case series were included. Articles not available in full text, nonhuman studies, and those not addressing clinical presentation were excluded. The summary of the reviewed literature is documented in Table 2.

**Table 2:** Summary of reviewed literature RLQ: right lower quadrant; RUQ: right upper quadrant; LUQ: left upper quadrant; LLQ: left lower quadrant

Study	DOI	Study type	Population	Atypical features	Diagnostic challenge	Outcome
Mohebbi et al. [28]	N/A	Case report	Middle-aged (50F)	RUQ pain mimicking cholecystitis	Ultrasound nondiagnostic	No complications
Ong and Venkatesh [29]	10.3748/wjg.15.3576	Case series + literature review	Young adults (4 patients; 30F, 31F, 34M, 27M)	RUQ/flank pain, atypical radiation	Misdiagnosed as biliary disease	Perforation, abscesses
Ball and Privitera [30]	10.1136/bcr-2013-009454	Case report + literature review	Middle-aged F	RUQ pain (subhepatic appendix)	Misdiagnosed as biliary disease	Ileus
Beh and Ongso [31]	10.1093/jscr/rjy081	Case report + literature review	Young adult (37M)	RUQ pain (renal agenesis)	Misdiagnosed as biliary disease	No complications
Hafiz et al. [32]	10.1053/j.gastro.2016.12.042	Clinical vignette	Young adult (32M)	Chronic RUQ pain	Normal initial labs/imaging	Symptom resolution post-op
Chao et al. [19]	10.7759/cureus.40772	Case report + literature review	Elderly (71M)	Chronic RUQ pain	Normal initial labs/imaging	No complications
Ferrie et al. [33]	10.7759/cureus.93826	Case report + literature review	Young adult (31F)	Chronic RUQ pain (retrocecal appendix)	Normal initial labs/imaging	No complications
Pastore et al. [34]	10.3122/jabfm.19.6.621	Case series + literature review	Young adults (22F, 39F); pregnant women	RLQ pain with pregnancy; nonspecific symptoms	Pregnancy-related	Fetal loss (1 case), otherwise recovery
Hou et al. [35]	10.1016/S1726-4901(09)70101-7	Case report	Adults (2 patients, 48M and 58F)	LLQ pain	Delayed/missed diagnosis	Rupture, abscess
Akbulut et al. [36]	10.3748/wjg.v16.i44.5598	Case series + literature review	Case: 25F; Review: 95 cases (ages 8-82)	LLQ pain due to situs inversus; variable pain locations	Atypical anatomy/location	Case: no complications; review: occasional perforation and abscess
Akbulut et al. [37]	10.1007/s11605-010-1210-2	Case series + literature review	Cases: 24M and 28M; Review: 63 cases (ages 8-82)	Variable abdominal pain locations (LLQ, LUQ, RLQ)	Historical/general	Some cases with perforation; case patients uncomplicated
Welte and Grosso [38]	10.1186/1752-1947-1-92	Case report + literature review	Middle-aged (46M)	LLQ pain (malrotation)	Mimic of other pathology	No complications
Shekhar et al. [18]	10.1016/j.ijscr.2015.06.036	Case report + literature review	Pediatric (10M)	LLQ pain (malrotation)	Ultrasound nondiagnostic	Perforated appendicitis
Chuang et al. [39]	10.1007/s12262-014-1200-9	Case report + literature review	Elderly (77F)	LLQ pain (malrotation)	Incidental finding	No complications
Aassouani et al. [40]	10.1016/j.radcr.2022.07.009	Case report + literature review	Pediatric (10M)	LLQ pain (malrotation)	Ultrasound nondiagnostic	No complications
Hu et al. [41]	10.3389/fsurg.2022.896116	Case report + literature review	Young adult (39M)	LLQ pain	Delayed/missed diagnosis	No complications
Tawk et al. [42]	10.1016/j.ijscr.2012.05.002	Case report + literature review	Adolescent (15F)	LUQ pain	Ultrasound nondiagnostic	No complications
Chuang et al. [43]	10.5812/iranjradiol.6326	Case report + literature review	Middle-aged (50M)	LUQ pain (malrotation)	Normal initial labs/imaging	No complications
Ozturk et al. [44]	10.5152/jemcr.2018.2085	Case report + literature review	Young adult (33M)	LUQ pain	Atypical anatomy/location	No complications
Lupiañez-Merly et al. [45]	10.12659/AJCR.908276	Case report + literature review	Adolescent (13F)	LUQ pain (malrotation)	Delayed/missed diagnosis	No complications
Hildenbrandt et al. [46]	10.7759/cureus.100026	Case report + literature review	Young adult (21F)	LUQ pain	Delayed/missed diagnosis	No complications

From the reviewed literature, atypical presentations of appendicitis were reported across a broad age range, from age 8 to 82. Although certain comorbidities such as diabetes mellitus, hypertension, dyslipidemia, and pregnancy were noted in a handful of cases, the vast majority of cases involved otherwise healthy individuals. A common theme across the studied cases was the finding of underlying anatomic variations, including intestinal malrotation, situs inversus totalis, subhepatic appendix, and renal agenesis. These variations were not isolated to one age range or sex, and the vast majority remained undiagnosed until imaging or surgical intervention was pursued. Of note, pregnant patients with typical appendicitis and those with congenital anatomical abnormalities appeared particularly complex to diagnose.

The most common clinical finding across all cases was abdominal pain outside the RLQ. Some patients presented with RUQ pain, often mimicking biliary pathologies such as cholecystitis. Patients with intestinal malrotation or situs inversus totalis often presented with left upper quadrant pain. Regardless of the localized site of pain, the vast majority of patients presented with other nonspecific symptoms, including nausea, vomiting, fever, and anorexia. The presence of nonspecific symptoms and atypical abdominal pain often led to diagnostic testing appropriate for conditions other than appendicitis, as the classic features of localized RLQ tenderness and migration of pain were absent. Certain cases also demonstrated recurrent or chronic pain patterns rather than acute, further complicating recognition and diagnosis of appendicitis.

The atypical presentations illustrated in all cases were consistently associated with incorrect initial diagnosis and ultimately delayed diagnosis. Some of the common misdiagnoses included acute cholecystitis, pyelonephritis, urinary tract infection, pelvic inflammatory disease, and gastroenteritis. During physical exams and initial workups, laboratory findings were often variable, and ultrasound frequently failed to detect the pathology, particularly in cases of anatomical variation. Some patients demonstrated leukocytosis, elevated erythrocyte sedimentation rate, and elevated C-reactive protein, while others had normal inflammatory markers. Following delayed diagnoses and misdiagnoses, CT proved to be the most reliable diagnostic modality, often providing the first definitive diagnosis. Several studies emphasized that reliance on classic signs, symptoms, and clinical scoring systems, such as the Alvarado score, for the diagnosis of appendicitis was unreliable in these atypical cases. In some cases, the diagnosis was made only during surgical intervention, further highlighting the limitations of standard diagnostic approaches.

In all cases of delayed diagnosis, there was an increased risk of potentially life-threatening complications, including perforated appendicitis and peritonitis. Other documented complications include intra-abdominal abscess formation, formation of dense adhesions, and, in some cases, the need for more extensive surgical interventions such as hemicolectomies. The most commonly observed complication was appendiceal perforation. The groups that appeared to be most vulnerable to life-threatening complications included pediatric patients and those with significant delays in diagnosis. However, when the diagnosis was made promptly, often following CT imaging, outcomes were generally favorable with uncomplicated recoveries following appendectomy.

In summary, atypical appendicitis frequently leads to misdiagnosis and therefore, delayed treatment, often due to variable and noncharacteristic symptoms and nonspecific clinical findings. These delays can be incredibly harmful, as they significantly increase the risk of perforation and other complications. These cases underscore the importance of early imaging and maintaining a high index of suspicion in patients with unexplained abdominal pain.

Despite numerous case reports describing atypical presentations of appendicitis, the existing literature is limited to single case studies and small case series. This significantly limits the generalizability of findings. This lack of broad application from limited literature, coupled with the unreliability of traditional diagnostic tools, severely limits the ability to establish a standardized diagnostic evaluation for patients presenting with atypical abdominal pain. Additionally, underlying anatomic variations such as intestinal malrotation and abnormal appendiceal positioning are frequently underrecognized contributors to atypical presentations of appendicitis. Collectively, these gaps highlight the need for increased awareness and more structured diagnostic approaches for atypical appendicitis.

Because it discusses a single patient, this report has limited generalizability. However, as it highlights the diagnostic challenges regarding RUQ appendicitis, which aligns with the current literature, this report may guide diagnostic decision-making in these rare cases and facilitate the reduction of undesired outcomes due to delayed diagnosis and treatment.

## Conclusions

This case report highlights the diagnostic and surgical challenges of perforated subhepatic appendicitis, an anatomical variant that mimics hepatobiliary pathology. The patient's RUQ pain with radiation to the back and shoulder and gastrointestinal symptoms appropriately prompted evaluation for hepatobiliary pathology. A negative ultrasound necessitated CT imaging, which was essential in identifying an inflamed appendix in an atypical location. Conversion from a laparoscopic to an open appendectomy was required due to a subhepatic cecum, retroperitoneal adhesions, cecal perforations, and intraoperative bleeding, emphasizing the importance of a carefully tailored surgical approach based on anatomical variation.

This case contributes to the literature by presenting the diagnostic pitfalls and approach regarding subhepatic appendicitis complicated by perforation, though it is limited by its single-center, single-patient nature. Further research with prospective registries or multicenter data collection for atypical appendicitis is needed to advance diagnostic accuracy and patient care. In patients with unexplained abdominal pain and inconclusive initial work-up, clinicians should keep appendicitis on their differential, regardless of pain location. Reducing morbidity in these cases relies on early CT imaging, consideration of anatomical variants, and preparedness to adjust the surgical approach.
